# Adiponectin receptor-1 expression is associated with good prognosis in gastric cancer

**DOI:** 10.1186/1756-9966-30-107

**Published:** 2011-11-11

**Authors:** Tomoya Tsukada, Sachio Fushida, Shinichi Harada, Shiroh Terai, Yasumichi Yagi, Jun Kinoshita, Katsunobu Oyama, Hidehiro Tajima, Hideto Fujita, Itasu Ninomiya, Takashi Fujimura, Tetsuo Ohta

**Affiliations:** 1 Department of Gastroenterological Surgery, Division of Cancer Medicine, Graduate School of Medical Science, Kanazawa University, 13-1 Takara-machi, Kanazawa, Ishikawa 920-8641, Japan; 2 Center for Biomedical Research and Education, School of Medicine, Kanazawa University, 13-1 Takara-machi, Kanazawa, Ishikawa 920-8641, Japan

**Keywords:** Adiponectin, AdipoR1, AdipoR2, gastric cancer, survival

## Abstract

**Background:**

Adiponectin is inversely related to BMI, positively correlates with insulin sensitivity, and has anti-atherogenic effects. In recent years, adiponectin has been well studied in the field of oncology. Adiponectin has been shown to have antiproliferative effects on gastric cancer, and adiponectin expression is inversely correlated with clinical staging of the disease. However, no studies have reported the correlation between serum adiponectin and receptor expression with disease progression.

**Methods:**

In this study, we evaluated expression levels of 2 adiponectin receptors--AdipoR1 and AdipoR2--and attempted to correlate their expression with prognosis in gastric cancer patients. AdipoR1 and AdipoR2 expression in gastric cancer cell lines (MKN45, TMK-1, NUGC3, and NUGC4) was evaluated by western blotting analysis, and the antiproliferative potential of adiponectin was examined in vitro. Serum adiponectin levels were evaluated in 100 gastric cancer patients, and the expression of AdipoR1 and AdipoR2 was assessed by immunohistochemical staining.

**Results:**

MKN45 and NUGC3 expressed higher levels of AdipoR1 compared to NUGC4, even though there was no significance in AdipoR2 expression. The antiproliferative effect of adiponectin was confirmed in MKN45 and NUGC3 at 10 μg/ml. No significant associations were observed between serum adiponectin levels and clinicopathological characteristics, but lymphatic metastasis and peritoneal dissemination were significantly higher in the negative AdipoR1 immunostaining group (24/32, *p *= 0.013 and 9/32, *p *= 0.042, respectively) compared to the positive AdipoR1 group (lymphatic metastasis, 33/68; peritoneal dissemination, 8/68). On the other hand, AdipoR2 expression was only associated with histopathological type (*p *= 0.001). In survival analysis, the AdipoR1 positive staining group had significantly longer survival rates than the negative staining group (*p *= 0.01). However, multivariate analysis indicated that AdipoR1 was not an independent prognostic factor on patient's survival on gastric cancer.

**Conclusions:**

In gastric cancer, adiponectin has the possibility to be involved in cell growth suppression via AdipoR1. The presence of AdipoR1 could be a novel anticancer therapeutic target in gastric cancer.

## Background

As the number of obese patients increases, there is growing interest in cytokines secreted by adipocytes. Human adiponectin (also known as *Acrp30 *[1] or *AdipoQ *[2]) is a 25-kDa adipocytokine composed of 247 amino acids; adiponectin is highly and specifically expressed in differentiated adipocytes and circulates at a concentration of 5-10 μg/ml in the blood stream [1-5].

Serum adiponectin levels correlate with insulin sensitivity and lipid metabolism [6,7]. Many studies have reported that adiponectin is related to obesity [8], metabolic syndrome [9,10], type 2 diabetes mellitus [11-13], and arteriosclerosis [14,15]. In addition, weight reduction increases adiponectin levels in obese patients [16]. Recent studies have shown that decreased plasma adiponectin levels significantly correlate with the risk of various cancers such as esophageal [17], colorectal [18], breast [19], endometrial [20], prostate [21], renal cell [22], and gastric cancer [23]. However, the role of adiponectin in cancer etiology is not yet fully understood. Although adiponectin may provide indirect protection against carcinogenesis by affecting insulin sensitivity and inflammatory states, it has direct anti-carcinogenic effects through the AMP-activated protein kinase (AMPK) system. Activated AMPK plays an important role in the regulation of growth arrest and apoptosis by stimulating p53 and p21 [24]. Moreover, independent of AMPK activation, adiponectin decreases production of reactive oxygen species (ROS) [25], which may result in decreased activation of mitogen-activated-protein-kinase (MAPK) [26] and subsequently results in inhibition of cell proliferation.

The adiponectin receptor exists in 2 isoforms: adiponectin receptor 1 (AdipoR1), which is abundantly expressed in skeletal muscle, and adiponectin receptor 2 (AdipoR2), which is predominantly expressed in skeletal muscle and the liver [27]. The expression of these receptors has been reported in gastric cancer cell lines, and adiponectin has been shown to inhibit proliferation and peritoneal dissemination through AdipoR1/R2 activation on gastric cancer cells [28]. However, the correlation between AdipoR1 or AdipoR2 expression and overall survival rate, and the clinical importance of these receptors remain unclear. In this study, we analyzed the correlation between serum adiponectin levels, expression of AdipoR1/R2, and clinicopathological characteristics as well as overall patient survival in gastric cancer.

## Methods

### Reagents and cell lines

Recombinant human adiponectin was purchased from R&D Systems, (Minneapolis, MN, USA), reconstituted in phosphate-buffered saline (PBS) at appropriate concentrations and stored at 4°C until use.

Human gastric cancer cell lines, TMK-1 (poorly differentiated adenocarcinoma) and MKN45 (poorly differentiated adenocarcinoma) were obtained from the American Type Culture Collection (Rockville, MD, USA), NUGC3 (poorly differentiated adenocarcinoma) and NUGC4 (signet ring cell carcinoma) were obtained from the Japanese Collection of Research Bioresources (National Institute of Health Sciences, Tokyo, Japan). The culture medium for cells was RPMI 1640 (Gibco, Invitrogen, Tokyo, Japan) supplemented with 10% heat-inactivated fetal bovine serum (Nichirei Bioscience Inc., Tokyo, Japan), 100 IU/ml penicillin, 100 mg/ml streptomycin (Gibco), and 2 mM glutamine (Nissui Pharmaceutical Co., Ltd., Tokyo, Japan). Cell lines were seeded in 75-cm^2 ^dish flasks (Becton Dickinson, Tokyo, Japan) and cultured in 10 mL of medium at 37°C in a humidified atmosphere of 5% CO_2 _in air. Cells were grown to confluence and harvested by trypsinization with 0.25% trypsin/EDTA (Gibco) and suspended in culture medium before use.

### Western blotting

Immunoblot analysis was performed as described previously [29]. Cells were lysed in RIPA buffer (50 mmol/l pH 8.0 Tris-HCl, 150 mmol/l sodium chloride, 0.5 w/v% sodium deoxycholate, 0.1 w/v% sodium dodecyl sulfate, and 1.0 w/v% NP-40 substitute) (Wako, Tokyo, Japan) containing 1% protease inhibitor cocktail (Sigma-Aldrich, St. Louis, MO, USA). The protein concentration of each sample was measured using a BCA protein assay kit (Pierce Biotechnology, Rockford, IL, USA). Whole-cell lysates were prepared in denaturing SDS sample buffer and subjected to SDS-PAGE (ATTO Co. Ltd., Tokyo, Japan). Proteins were transferred to PVDF membranes (Bio-Rad Laboratories, Hercules, CA, USA) and then blocked with commercial gradient buffer (EzBlock; Atto Corporation, Tokyo, Japan) at room temperature for 30 min. The immunoblots were visualized using an ECL Plus kit (GE Healthcare UK Ltd., Tokyo, Japan). The antibody-antigen complex was detected using an ECL Western-Blotting detection kit (GE Healthcare) and the Light-Capture system (ATTO), and then quantified using the CS analyzer program (ATTO). All experiments were repeated three times. We used the following primary antibodies: anti-AdipoR1 antibody (C-14, goat polyclonal IgG, diluted 1:100; Santa Cruz Biotechnology Inc., Santa Cruz, CA, USA), anti-AdipoR2 (C-12, goat polyclonal IgG, diluted 1:100; Santa Cruz), and anti-β-actin (AC-15, mouse monoclonal IgG, diluted 1:10,000; Sigma-Aldrich).

### Cell growth assay

The viability of gastric cancer cell lines treated with adiponectin was determined by standard 3-(4, 5-dimethylthiazol-2-yl)-2, 5-diphenyltetrazolium bromide (MTT) assay. Cell were seeded at 5 × 10^3 ^cells per well in 96-well plates and incubated overnight at 37°C. After incubation, the supernatant was discarded and replaced with fresh serum-free culture medium. Adiponectin was dissolved in PBS and added to the cell culture medium at various concentrations (0, 0.1, 1, 5, or 10 μg/ml). At 48 h after exposure to adiponectin, the supernatant was discarded, and MTT solution was added to each well (500 μg/mL, final concentrations) and incubated at 37°C for 3 h. The supernatant was removed, and 150 μL of dimethylsulfoxide (DMSO: Wako, Japan) was added. The absorbance of the solution was read at a wavelength of 540 nm using a microplate reader (BIO-RAD550; BIO-RAD, Tokyo, Japan). The percentage inhibition was determined by comparing the cell density of the drug-treated cells with that of untreated controls. All experiments were repeated at least 3 times.

### Specimens and blood samples

We evaluated 100 patients with gastric cancer (cases) who were treated with curative gastrectomy and standard lymph node dissection at the Gastroenterological Surgery Department, Kanazawa University Hospital, Ishikawa, from 2002 to 2009. The study was approved by the ethics committee of Kanazawa University, and informed consent was obtained from each patient before enrollment in this study. All resected primary tumors and regional lymph nodes were histologically evaluated by H&E staining according to the Japanese Classification of Gastric Carcinoma [30]. A fasting morning blood sample was obtained for the adiponectin assay from each patient after admission into the study. Samples were also obtained from 10 healthy volunteer controls. Weight and height of each patient was recorded by medical staff. BMI was calculated as weight in kilograms divided by height in square meters. Medical staff measured all data.

### Serum adiponectin measurement

All blood samples were immediately separated by centrifugation and stored at -80°C until use. A quantitative sandwich enzyme-linked immunosorbent assay technique with a Quantikine human adiponectin immunoassay kit (R&D Systems, Inc., Minneapolis, NM, USA) was used in accordance with the manufacturer's instructions. All experiments were performed in triplicate.

### Immunohistochemical staining

All surgically obtained specimens were fixed in 10% neutral buffered formalin, embedded in paraffin, and cut into 4-μm-thick serial sections. In brief, the slides were immersed in methanol containing 0.3% H_2_O_2 _for 30 min, blocked with 3.3% normal goat serum in PBS, and incubated with the anti-AdipoR1 antibody (C-14, goat polyclonal IgG, diluted 1:100; Santa Cruz Biotechnology Inc., Santa Cruz, CA, USA) and anti-AdipoR2 (C-12, goat polyclonal IgG, diluted 1:100; Santa Cruz) at 4°C overnight. After the sections were washed in PBS, immunoreactivity was visualized by EnVision reagent (Dako Co., Kyoto, Japan). Slides were examined under low power (×40) to identify the brown staining precipitates within the cytoplasm of cancer cells. Sections that showed same or higher staining than that of the normal gastric mucosa and more than 10% of cancerous tissue stained under a ×100 field were considered positive samples.

### Statistical analysis

Values are expressed as means ± standard error (SE). Differences in the cell growth assay were determined by one-way analysis of variance (ANOVA). The relationship between serum adiponectin level and BMI or clinical stage of gastric cancer was evaluated using the Mann-Whitney U test. Fisher exact and χ^2 ^test were used to evaluate statistical correlations between plasma adiponectin levels, the expression of AdipoR1 or AdipoR2 in cancerous tissues, and various clinicopathological variables. Overall survival rates were estimated using the Kaplan-Meier method, and a log-rank test was used to compare results between survival time and AdipoR1 or AdipoR2 immunohistochemical expression. The influence of various clinicopathological factors, including AdipoRs expression, on survival was assessed by the Cox proportional hazards model (multivariate analysis) using backward-LR methods. All statistical analyses were performed using the computer software package SPSS 10.0 (SPSS Inc., Chicago, IL, USA). Significance was defined as *p *< 0.05.

## Results

### Expression of AdipoR1/R2 and effect of adiponectin on gastric cancer cells

**Figure 1:**
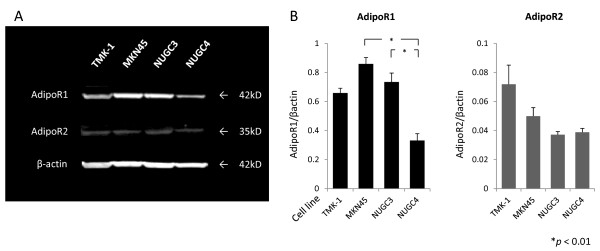
**The expression of AdipoR1 and AdipoR2 in human gastric cancer cell lines**. (A) Western blotting analysis for AdipoR1 (42 kD), AdipoR2 (35 kD), and β-actin (42 kD) in human gastric cancer cell lines. (B) Densitometric analysis were performed. The results are mean ± SE values of 3 different experiments.

To determine the expression of AdipoR1/R2 in gastric cancer cell lines, western blotting analysis was performed. As shown in Figure 1A, AdipoR1/R2 were positively detected in cell lines, and compared with NUGC4, MKN45 and NUGC3 had higher expression of AdipoR1. On the other hand, no significant differences were observed in expression of AdipoR2 (Figure 1B).

**Figure 2:**
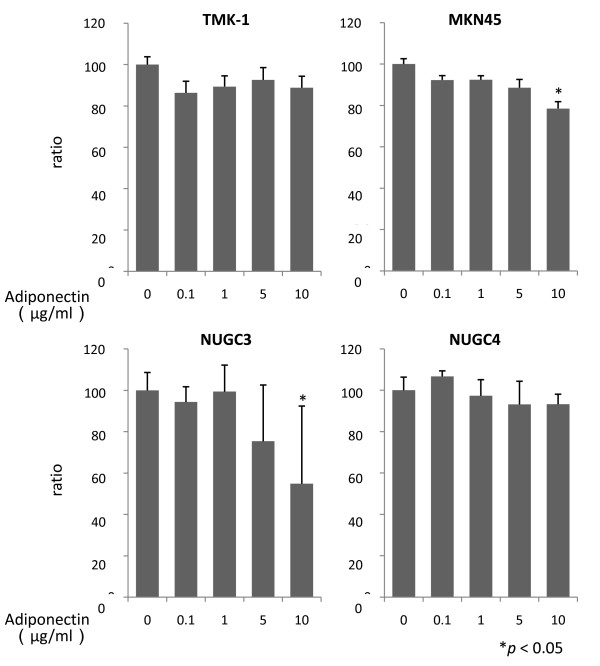
**The effect of adiponectin on cell proliferation**. Cell viability was assessed after 48-h exposure to a single dose of adiponectin (0, 0.1, 1, 5, or 10 μg/ml) in serum-free medium. The results are mean ± SE values of 3 different experiments.

In MKN45 and NUGC3, adiponectin significantly suppressed proliferation at 10 μg/ml (78.5% ± 3.3%, 54.9% ± 37.5%, respectively, *p *< 0.05). In contrast, NUGC4 and TMK-1 were slightly suppressed after 48 h exposure of adiponectin, but the effect was not significant even at a concentration of 10 μg/ml (Figure 2).

### Serum adiponectin and clinicopathological characteristics

**Figure 3:**
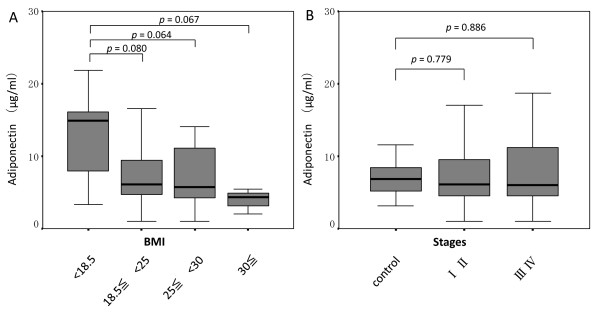
**Correlation between serum adiponectin level and body mass index or tumor stages**. Correlation between serum adiponectin level and body mass index (A) or tumor stages (B) in gastric cancer. Box plots show interquartile range (box), median (thick line), and range (thin line).

As shown in Figure 3, no significant differences were observed between serum adiponectin and BMI in gastric cancer patients. However, adiponectin concentrations showed a tendency to decrease gradually with an increase in BMI (Figure 3A). Compared with the control group, no significant differences in adiponectin were observed between tumor stages (Figure 3B).

The mean value of serum adiponectin in the control group was 7.0 ± 2.4 μg/ml. Therefore, we divided the patients into low (n = 39) and high (n = 61) groups using a cutoff value of 7.0, and clinicopathological characteristics were compared between the 2 groups (Table 1). No significant differences were observed in age, BMI, macroscopic tumor type, depth of tumor invasion, histopathological type, lymphatic invasion, venous invasion, lymphatic metastasis, peritoneal dissemination, hematogenous metastasis, or tumor stages between the 2 groups. Forty-six (69.7%) of 66 male patients were categorized in the low group, whereas only 15 (44.1%) of 34 female patients were categorized in this group.

**Table 1:** Correlation between serum adiponectin level and clinicopathological characteristics in gastric cancer patients.

	Adiponectin high group (n = 39)	Adiponectin low group (n = 61)	p value
Age (y)	63.5 ± 12.1	60.6 ± 13.2	0.275
Gender			
Male	20	46	0.013
Female	19	15	
BMI	22.1 ± 3.6	23.4 ± 3.9	0.079
Macroscopic type			
Elevated	5	6	0.642
Depressed/flat	34	55	
Depth of invasion			
T1	15	31	0.227
T2, T3 and T4	24	30	
Histological type			
differentiated	17	22	0.558
undifferentiated	23	38	
Lymphatic invasion			
positive	32	42	0.142
negative	7	19	
Venous invasion			
positive	22	33	0.821
negative	17	28	
Lymphatic metastasis			
positive	23	34	0.750
negative	16	27	
Peritoneal dissemination			
positive	9	8	0.196
negative	30	53	
Hematogenous metastasis			
positive	1	3	0.558
negative	38	58	
Stage			
I and II	26	41	0.910
III and IV	13	20	

### AdipoR1/R2 expression in gastric cancer

**Figure 4:**
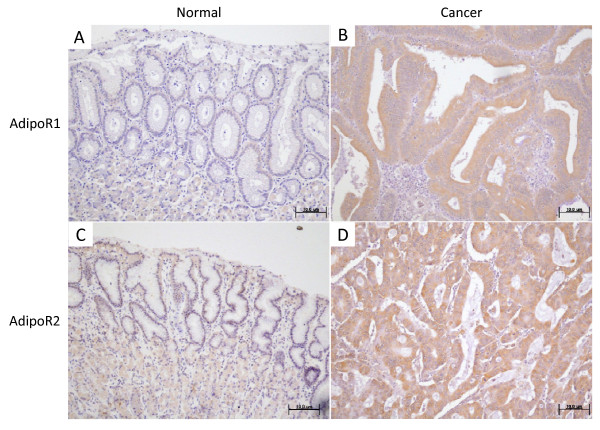
**Representative photomicrographs**. Representative photomicrographs of immunohistochemical staining of AdipoR1 (A, normal mucosa; B, cancer tissue) and AdipoR2 (C, normal mucosa; D, cancer tissue). AdipoR1 and AdipoR2 were expressed in normal gastric mucosa in the cytoplasm as well as in the cell membrane. In gastric cancer tissues, higher intensity of immunostaining compared to normal mucosa was considered positive. Original magnification, ×100.

The protein expression of AdipoR1 and AdipoR2 was confirmed by immunostaining of surgically resected gastric cancer tissue specimens (Figure 4). AdipoR1 and AdipoR2 were positively detected in the cytoplasm as well as the cell membrane of cancer cells. In contrast, normal gastric epithelial cells did not show significant immunoreactivity for either AdipoR1 or AdipoR2. In some parietal cells of normal gastric mucosa, slight reactivity was observed in AdipoR2 expression. This was in accordance with the findings of Ishikawa et al [28].

AdipoR1 expression was significantly associated with histopathological type (*p *= 0.011) (Table 2). In addition, negative AdipoR1 immunostaining was significantly higher in patients with lymphatic metastasis (*p *= 0.013; Table 2) and peritoneal dissemination (*p *= 0.042; Table 2). On the other hand, AdipoR2 expression was also associated with the histopathological type (*p *= 0.001; Table 3). However, no significant differences were observed in other clinicopathological characteristics (Table 3).

**Table 2:** Expression of AdipoR1 and clinicopathological characteristics in gastric cancer patients.

	AdipoR1 positive (n = 68)	AdipoR1 negative (n = 32)	p value
Age (y)	62.7 ± 11.0	59.7 ± 16.0	0.284
Gender			
Male	44	22	0.690
Female	24	10	
BMI	23.3 ± 4.0	22.1 ± 3.4	0.161
Serum adiponectin (μg/ml)	7.4 ± 5.0	8.9 ± 6.1	0.193
Macroscopic type			
Elevated	8	3	0.722
Depressed/flat	60	29	
Depth of invasion			
T1	34	12	0.242
T2, T3 and T4	34	20	
Histological type			
differentiated	33	7	0.011
undifferentiated	35	25	
Lymphatic invasion			
positive	49	25	0.519
negative	19	7	
Venous invasion			
positive	37	18	0.863
negative	31	14	
Lymphatic metastasis			
positive	33	24	0.013
negative	35	8	
Peritoneal dissemination			
positive	8	9	0.042
negative	60	23	
Stage			
I and II	49	18	0.171
III and IV	19	14	

**Table 3:** Expression of AdipoR2 and clinicopathological characteristics in gastric cancer patients.

	AdipoR2 positive (n = 72)	AdipoR2 negative (n = 28)	p value
Age (y)	62.1 ± 12.3	60.7 ± 14.2	0.624
Gender			
Male	52	14	0.035
Female	20	14	
BMI	22.9 ± 3.9	23.1 ± 3.8	0.719
Serum adiponectin (μg/ml)	7.9 ± 5.5	8.0 ± 5.1	0.968
Macroscopic type			
Elevated	10	1	0.139
Depressed/flat	62	27	
Depth of invasion			
T1	33	13	0.957
T2, T3 and T4	39	15	
Histological type			
differentiated	36	4	0.001
undifferentiated	36	24	
Lymphatic invasion			
positive	55	19	0.382
negative	17	9	
Venous invasion			
positive	41	14	0.531
negative	31	14	
Lymphatic metastasis			
positive	42	15	0.666
negative	30	13	
Peritoneal dissemination			
positive	11	6	0.462
negative	61	22	
Stage			
I and II	46	21	0.289
III and IV	26	7	

### Survival analysis

**Figure 5:**
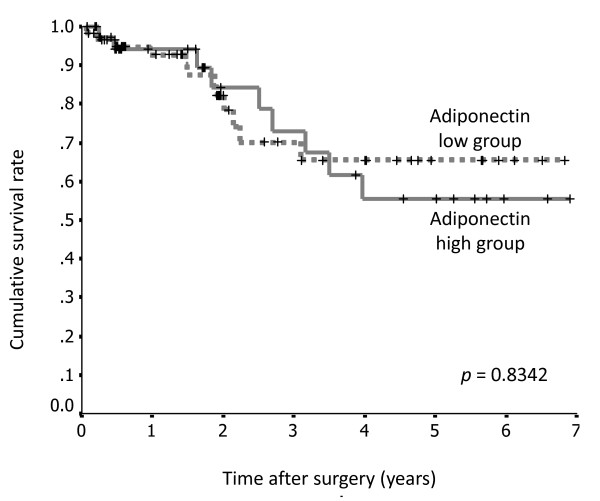
**Survival curves for 100 patients with gastric cancer after surgery, according to serum adiponectin level**. There was no significant difference between the high serum adiponectin level group (n = 61) and the low serum adiponectin level group (n = 39).

Survival rates according to serum adiponectin levels, the presence or absence of AdipoR1 expression, and AdipoR2 expression were assessed using the Kaplan-Meier method. There were no significant differences in survival rate between the groups with high and low serum adiponectin levels (*p *= 0.8342; Figure 5).

**Figure 6:**
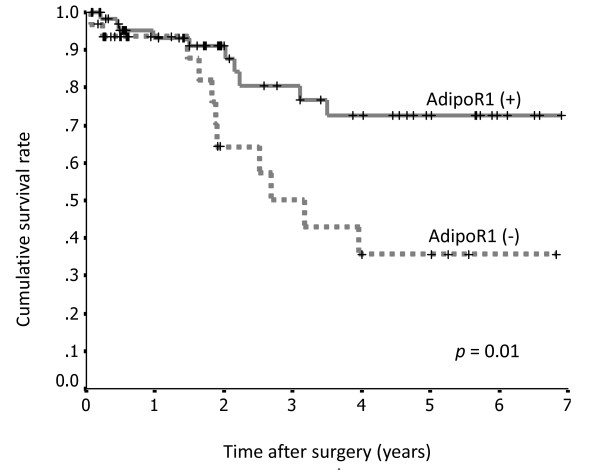
**Survival curves for 100 patients with gastric cancer after surgery, according to AdipoR1 expression**. The survival rate of patients with gastric cancer positive for AdipoR1 expression (n = 68) was significantly greater than that of patients negative for AdipoR1 (n = 32).

**Figure 7:**
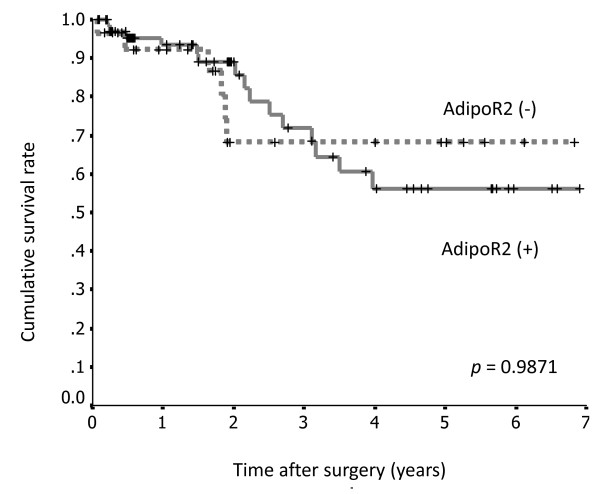
**Survival curves for 100 patients with gastric cancer after surgery, according to AdipoR2 expression**. There was no significant difference between the AdipoR2-positive group (n = 72) and the AdipoR2-negative group (n = 28).

Patients with positive AdipoR1 staining had a significantly longer survival rate than those with negative staining (*p *= 0.01; Figure 6), whereas there were no significant differences in AdipoR2 expression between these 2 groups (*p *= 0.9871; Figure 7).

Multivariate analysis indicated that only the peritoneal dissemination was an independent prognostic factor on patient's survival (*p *= 0.001; Table 4).

**Table 4:** Multivariate analysis for 100 patients with gastric cancer.

Variable	B	SE	Exp (B)	p value
Histological type	0.394	0.552	1.482	0.476
Peritoneal dissemination	1.700	0.465	5.474	0.001
AdipoR1 expression	0.718	0.447	2.051	0.108

## Discussion

Adiponectin, which belongs to the complement 1q family, is composed of an N-terminal collagen-like sequence and a C-terminal globular region, is well studied in the field of oncology, and its expression is inversely related to weight gain [31]. Ishikawa et al. reported that a low serum adiponectin level was associated with an increased risk of gastric cancer, although BMI did not differ significantly [23]. In our study, we were also unable to detected significant differences with respect to serum adiponectin levels and BMI. However, visceral fat predominantly correlates with serum adiponectin levels [32], and BMI cannot be used to distinguish fat distribution (for example, subcutaneous fat versus visceral fat); this may be the reason for the failure to find a significant correlation between the 2 parameters. In addition, a correlation was not observed between the amounts of serum adiponectin and clinicopathological factors or prognosis in gastric cancer. Ishikawa et al. indicated a tendency of an inverse correlation between tumor stage and serum adiponectin levels, but significant difference was not demonstrated in the current study. With respect to clinicopathological factors, there were significant differences in adiponectin levels according to tumor location and differentiation [23]. Seker et al. also reported significant difference between degrees of tumor differentiations and adiponectin levels [33]. Gastric cancer patients tend to be cachexic with the progression of primary disease, and this can result in high serum adiponectin levels [34]. Consequently, it is difficult to elucidate the clinicopathological significance of adiponectin in gastroenterological cancer patients because of the aforementioned contradictory relationship [35]. As a result of this lack of significant difference between the clinicopathological factors and serum adiponectin levels, it is presumed that serum adiponectin levels do not contribute to prolonged survival in gastric cancer patients.

Generally, it is expected that receptor expression is more important than the amount of serum ligand, but no studies have addressed serum adiponectin and receptor expression levels.

Moreover, the expression of adiponectin receptors in gastric cancer cell lines has already been reported [28]. They also demonstrated that the inhibitory effects of adiponectin via AdipoR1 and AdipoR2 using specifically down-regulated experiments by siRNA. In their study, siRNA of adipoR1 strongly abolished the effects of adiponectin, although the effect of siRNA of adipoR2 was less prominent. In our examination, adiponectin led to growth inhibition in MKN45 and NUGC3. The two cell lines expressed AdipoR1 strongly, even though there were no significance in AdipoR2 expression. Therefore, it is likely that AdipoR1 plays an important role in cell proliferation. Although AdipoR1 and R2 are known as receptor subtypes, the relationship between gastric cancer and each subtype has not yet been clarified. Therefore, we evaluated the association between AdipoR expression and clinicopathological characteristics. The expression rates of both receptors were lower in histopathologically undifferentiated tumor types. However, the significant findings in our series indicate that the AdipoR1 expression-positive group showed lower lymphatic metastasis and peritoneal dissemination than the negative group. On the other hand, no clear associations were observed between AdipoR2 expression and any of the clinical characteristics that we evaluated. Otani et al. [36] reported that there are no significant associations between AdipoR1 mRNA levels and various pathological features in gastric cancer, whereas Barresi et al. reported longer overall survival in patients with positive AdipoR1/R2 expression [37]. Our clinical results reconfirm that AdipoR1 expression inversely correlates with tumor growth and might contributes to improvement of prognosis significantly, but not independently, in gastric cancer patients. However, expression of AdipoR2 does not affect prognosis, and there was no correlation between clinicopathological factors and AdipoR2 expression.

Adiponectin can exist as a full-length or a smaller, globular fragment. It has been proposed that the globular fragment is generated by proteolytic cleavage, and it has recently been shown that the cleavage of adiponectin by leukocyte elastase secreted from activated monocytes and/or neutrophils could be responsible for the generation of the globular adiponectin fragment [38]. On the other hand, AdipoR1 and AdipoR2 may form both homo- and heteromultimers. Scatchard plot analysis revealed that AdipoR1 is a receptor for globular adiponectin, whereas AdipoR2 is a receptor for the full-length form of adiponectin [39]. The ability of adiponectin to inhibit caspase-3 mediated cell death has been reported in various cells, including endothelial, neuroblastoma, and pancreatic β cells [40-42]. Park's group [43] demonstrated that globular adiponectin acting via AdipoR1 could protect mouse cardiomyocytes from apoptosis. Here, we show a cytostatic effect of adiponectin via AdipoR1, but the repression of cell proliferation via both AdipoR1- and AdipoR2-mediated AMPK has been also reported [44].

The improvement of prognosis in gastric cancer patients with positive AdipoR1 expression might be affected by organ protective effects from insulin resistance and inflammatory states rather than as a result of a direct antiproliferative effect via globular adiponectin.

## Conclusions

Our data suggest that adiponectin has antiproliferative potential; however, AdipoR1 plays a more important role in increased survival in gastric cancer patients. The mechanisms underlying the anti-tumor effects of adiponectin and the functional properties of AdipoR have not been fully elucidated. Although further research in this field is necessary, the presence of AdipoR1 could be a novel anticancer therapeutic target in gastric cancer.

## Competing interests

The authors declare that they have no competing interests.

## Authors' contributions

TT carried out most of experiments, participated in the design of the study, performed the statistical analysis, and drafted the manuscript. SF, SH, ST, and YY participated in the design of the study and helped draft the manuscript. JK, KO, HT, and HF assisted the experiments. IN, TF, and TO participated in the study design and coordination. All authors have read and approved the final manuscript.
